# The Effectiveness of Educational Mobile Messages for Assisting in the Prevention of Early Childhood Caries: Protocol for a Randomized Controlled Trial

**DOI:** 10.2196/13656

**Published:** 2019-09-03

**Authors:** Patricia Estefania Ayala Aguirre, Matheus Lotto, Anna Paola Strieder, Agnes Fátima Pereira Cruvinel, Thiago Cruvinel

**Affiliations:** 1 Department of Pediatric Dentistry, Orthodontics and Public Health Bauru School of Dentistry University of São Paulo Bauru Brazil; 2 Discipline of Public Health School of Medicine Federal University of Fronteira Sul Chapecó Brazil

**Keywords:** eHealth, dental caries, randomized controlled trial

## Abstract

**Background:**

In 2017, approximately 3.7 billion downloads of health apps were made on mobile phones and tablets. In this sense, a massive number of people could benefit by electronic mobile–based health interventions, making information available even with the lack of material and human resources. Hence, the use of electronic apps for dental education might be extremely useful for the prevention of early childhood caries (ECC).

**Objective:**

This study aims to evaluate the effectiveness of messages sent via mobile phones as an adjuvant method for the prevention of ECC.

**Methods:**

A single-blinded, randomized, and parallel-group clinical trial will be conducted with dyads of parents or caregivers and children aged between 36 and 60 months, recruited from kindergartens and schools of Bauru, São Paulo. The determination of sample size resulted in a total of 104 dyads of parents and children, considering a power of 80%, a significance level of 5%, and an attrition of 30%. This sample will be randomly assigned to test and control groups, being divided in 52 dyads per group according to the health literacy levels of parents and the age, gender, and oral health status of children. Every 2 weeks, only participants in the test group will receive messages via WhatsApp containing preventive and education-related ECC information. The dyads will visit the dentist every 3 months during a year for the assessment of primary outcomes (sugar consumption and the International Caries Detection and Assessment System, visible plaque, and community periodontal indices) and to receive dental care measures. Secondary outcomes (electronic health literacy and general perceived self-efficacy) will be determined only at baseline and after 12-month follow-up. The quality of randomization will be evaluated throughout the study, comparing the test and control groups systematically by Student t tests for continuous variables and chi-square tests for categorical variables. Listwise deletion method will be applied in cases of dropouts, if the missing values satisfy the criteria of missing completely at random; otherwise, multiple imputation data strategy will be conducted. The Kolmogorov-Smirnov and Levene tests will be used to determine the normality and homogeneity of data, respectively, which will indicate further statistical analyses for elucidating significant differences between groups (P<.05). A Student t test or Mann-Whitney U test will be employed for parametric or nonparametric analyses, respectively.

**Results:**

The project was funded in 2018, and enrollment was completed in August 2019. Allocation is currently under way and the first results are expected to be submitted for publication in 2020.

**Conclusions:**

The results will contribute to understanding the importance of educational mobile messages toward the adoption of healthy behaviors for the prevention of ECC in a given population.

**Trial Registration:**

Brazilian Registry of Clinical Trials Universal Trial Number U1111-1216-1393; http://www.ensaiosclinicos.gov.br/rg/RBR-2b6r7q/

**International Registered Report Identifier (IRRID):**

PRR1-10.2196/13656

## Introduction

### Background

The unprecedented spread of electronic technologies has facilitated the access of internet users to health information [1-3]. In 2017, approximately 3.7 billion downloads of mobile health apps were made to be used on cellphones and tablets [4]. In this sense, a large number of people could be benefited by health interventions using mobile devices, providing information on health care even in situations of low availability of material and human resources [5]. Currently, WhatsApp Messenger (WhatsApp Inc.) is one of the most popular mobile apps worldwide, with approximately 300 million users [6]. It connects people through free electronic messages, requiring only a Wi-Fi internet network [7,8]. WhatsApp Messenger has been shown to be a promising tool for the communication between patients and professionals, aiding in the spreading of health-related information [9].

In this context, the use of electronic apps for dental education might be extremely useful for the prevention of early childhood caries (ECC). It is defined as the presence of one or more decayed, lost, or restored surfaces found in deciduous teeth of children aged up to 71 months [10]. This disease affects about 621 million children, positioning it as the tenth most prevalent chronic disease out of 291 conditions of Global Burden of Disease Study [11]. ECC causes the impairment of mastication and speech, pain, psychological problems, and negative effects on the weight and growth of children [12-14]. In addition, approximately 94% of children diagnosed with ECC develop carious lesions in permanent dentition [15]. The identification of individual risk factors, parental counseling, and health promotion have played an important role in the prevention of ECC [16], contributing to the improvement of the quality of life of children and their families.

### Objectives

Therefore, new approaches are required to prevent ECC, aiming to improve the awareness about its consequences for deciduous and permanent dentitions, in addition to promoting the engagement of parents or caregivers with healthy behaviors toward the maintenance of oral health status of their children. In this sense, this study aims to assess the effectiveness of educational mobile messages sent via WhatsApp Messenger as an adjuvant method for the prevention of ECC in children of a given population. The null hypotheses for this study indicate that the educational mobile messages will not be effective for aiding in the control of dental plaque (H_0_), the decrease of sugar consumption (H_0_′), the maintenance of the International Caries Detection and Assessment System (ICDAS) indices (H_0_′′), and the improvement of parents’ electronic health (eHealth) literacy levels (H_0_′′′) after 12-month follow-up.

## Methods

### Overview

This study was reviewed and approved by the Council on Ethics in Human Research from the Bauru School of Dentistry (CAAE: 90563618.6.0000.5417), registered in the Brazilian Clinical Trials Registry (RBR-2b6r7q), and assigned with the universal trial number U1111-1216-1393. The peer review report is available in Multimedia Appendix 1.

### Study Design

**Figure 1:**
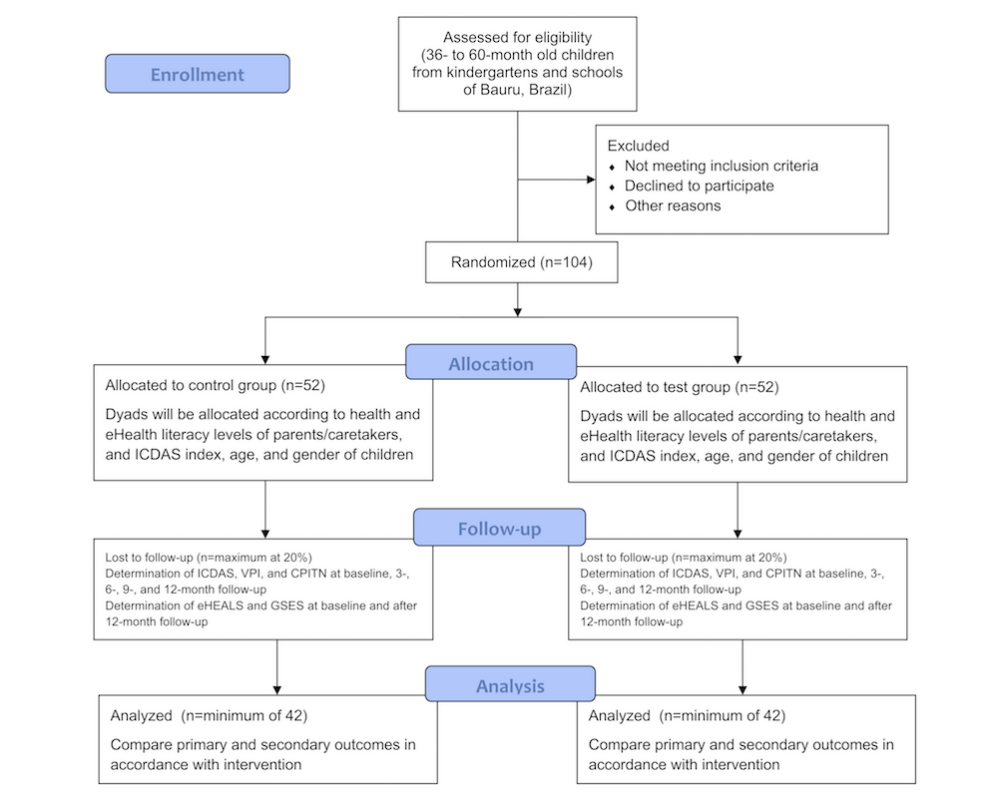
Study flowchart. CPITN: community periodontal index of treatment needs; eHealth: electronic health; eHEALS: The eHealth Literacy Scale; GSES: General Perceived Self-Efficacy Scale; ICDAS: International Caries Detection and Assessment System; VPI: visible plaque index.

This protocol describes a single-blinded, parallel, and randomized controlled trial (RCT) that will analyze the effectiveness of educational mobile messages as an adjuvant strategy for the prevention of ECC. The messages will be periodically sent via WhatsApp Messenger to parents or caregivers of children, who will be recruited from kindergartens and schools of Bauru, Brazil. The participants will be randomly allocated to 2 different groups (test and control). This RCT will be conducted according to the checklist and guidelines of the Consolidated Standards of Reporting Trials of Electronic and Mobile Health (CONSORT EHEALTH; beta version 1.5) [17]. Figure 1 depicts the synthesis of the design of this study.

### Eligibility Criteria

Dyads of parents and children will be recruited during visits to kindergartens and schools located in Bauru, Brazil, if satisfying the following inclusion criteria: children aged between 36 and 60 months; children at high risk of dental caries, according to the criteria described in the Caries Risk Assessment Form for 0- to 5-Year Olds [18]; children with a score ≤3, according to the criteria of ICDAS [19]; parents or caregivers with a mobile phone with internet access; parents or caregivers who accept to participate in all stages of research by signing a written informed consent form; and parents or caregivers who already have WhatsApp Messenger app installed on their smartphones, or those who agree to install it for the participation in the study.

The risk of dental caries will be based on the information collected during the anamnesis and clinical examination, which will be performed by a trained and calibrated dentist. Children will be examined in a dental clinic, using compressed air and artificial lighting, following the ICDAS criteria [19], the visible plaque index (VPI) [20], and the community periodontal index of treatment needs (CPITN) [21].

The oral hygiene level of children will be determined by the observation of visible plaque on the buccal dental surfaces of 6 deciduous teeth (#55, #53, #51, #71, #73, and #75) [20]. The periodontal health status of children will be assessed considering 3 parameters of analysis: presence of bleeding, detection of dental calculus, and depth of gingival sulcus [21]. According to Almeida et al [22], this study will classify deciduous teeth with 1 of 3 different scores: healthy (0), gingival bleeding (1), and presence of dental calculus (2).

### Electronic Health Literacy and Sociodemographic Questionnaire

The levels of eHealth literacy of parents or caregivers will be determined by the application of a previously validated Brazilian version of the eHealth Literacy Scale [23]. The eHealth Literacy Scale (eHEALS) will be applied by a trained professional, who provides the participants with a sheet containing 8 items related to skills needed to the adequate consumption of eHealth information. The answers of each item are arranged into a 5-point Likert scale, with options ranging from *completely agree* to *strongly disagree* [23]. The participants will be instructed to classify each item according to their own perception, achieving a total score varying from 8 to 40, with higher scores representing higher self-perceived eHealth literacy.

Finally, parents or caregivers will be invited to answer a questionnaire containing sociodemographic and child-related health information such as age, gender, race, and education level.

### International Caries Detection and Assessment System Training and Calibration

Overall, 2 investigators will be trained and calibrated for the use of ICDAS index according to the recommendations described below:

- An official trainer will make a 4-hour presentation, including images, discussions on ICDAS codes, and examination protocols.
- A total of 2 days of training will be given with the same number of teeth coded with ICDAS between 1 and 5, including the clinical examination of patients with decayed and extracted teeth.
- Examination findings will be reviewed to verify the repeatability of tests and differences in the interpretation until consensus among examiners.
- A total of 2 days of concordance evaluation will be performed, using at least 20 patients with dental caries lesions with ICDAS ranging from 1 to 5 (kappa>0.65) [19].

### Randomization

Parent and child dyads will be randomly assigned to test or control groups, according to the parameters of stratification, as follows: health and eHealth literacy levels of parents or caregivers and the ICDAS index, age, and gender of children. The randomization will be performed using the platform Randomization.com [24], through different block sizes for the allocation of participants. The blinding of allocations will be guaranteed by using closed and opaque envelopes, which will be maintained confidential by an independent researcher.

### Sample Size

The calculation of this sample size was performed using the Open Source Epidemiologic Statistics for Public Health, following the criteria and outcomes described by Zotti et al [25]. It resulted in a total of 104 dyads of parents and children, considering a power of 80%, a significance level of 5%, and an attrition of 30%.

### Intervention

Every 2 weeks, the parents or caregivers of test group will receive educational mobile messages related to the prevention of ECC via WhatsApp Messenger. These materials will be developed by the researchers from the insights, including main doubts, questions, and challenges, regarding the disease, collected from the focus groups, conducted previously with the participation of a sample of parents or caregivers who attended the Clinics of Bauru School of Dentistry. These text messages will be formulated with a simple language for a better understanding, which will include emoticons and short and direct videos. The participants will be instructed to activate the function *read receipts* to confirm their adherence and engagement with the study. The participants’ feedbacks related to the quality and utility of electronic messages for ECC prevention will be collected only after the 12-month follow-up, to avoid blinding biases.

All dyads of parents and children will be invited for dental appointments on a quarterly basis. The children will be examined by a trained and calibrated dental professional for the measurement of primary outcomes, receiving a subsequent dental prophylaxis. When necessary, fluoride varnish (Duraphat, Colgate) will be applied on dental demineralized surfaces (ICDAS=1 or 2), whereas a conventional glass ionomer cement (Ketac Molar Easymix, 3M) will be used for sealing localized enamel lesions (ICDAS=3). The investigators will not be informed about the origin of groups of participants, characterizing a single-blind study.

### Sugar Consumption

A questionnaire developed by Llena and Forner [26] to investigate the dietary habits of dental patients will be applied at baseline and after the 12-month follow-up to evaluate the influence of sugar consumption on the development of oral diseases. The different types of food are divided into 9 categories: (1) foods containing sticky sugars: dried fruit, candies containing sugar, jellies, jams, and sauces; (2) foods containing starch and sugar: cookies, cereals, and industrialized cakes; (3) candy without sugars; (4) milk and dairy products containing sugar: chocolate, yogurt, creams, ice creams, and flans; (5) milk and dairy products without sugar: pure milk, sugar-free yogurt, and cheese; (6) sugary beverages: juices and soft drinks; (7) fruits: fruits and juices; (8) semihydrolyzed starch-rich foods: potato chips, French fries, industrialized bread, and rolls; and (9) sugar-free foods: nuts, bread, pasta, and noodles.

### Study Outcome Measures

The primary outcomes will be related to the oral health status of children and the risk factors for ECC. For that, ICDAS, VPI, and CPITN indices will be determined for the recruitment of participants at baseline and 3-, 6-, 9-, and 12-month follow-ups.

The secondary outcomes will be related to the measurement of the influence of educative strategies on parents or caregivers, considering their patterns of sugar consumption, their level of eHealth literacy, and their individual perceptions about their own ability in executing specific activities. These data will be collected at baseline and after the 12-month follow-up using the questionnaire for sugar consumption [26], the instruments eHEALS [23], and General Perceived Self-Efficacy Scale (GSES) [27], respectively. In addition, the results of eHEALS will be applied for the recruitment of participants.

GSES measures the individual’s beliefs about their cognitive, motivational, behavioral, and affective skills needed to perform particular tasks. It comprises 10 questions with answers arranged in a 4-point Likert scale, with options ranging from *not at all true* through *exactly true*. The total score ranges between 10 and 40 points, with greater scores indicating higher overall perceived self-efficacy of individuals [27].

### Data Analysis

Statistical analysis will be performed using SPSS Statistics software 21.0 (IBM SPSS Statistics). The data will be presented with descriptive statistics, being examined for lost values, outliers, normality, and homogeneity. To investigate the quality of randomization, potential differences between the characteristics of participants of the test and control groups will be determined systematically, applying Student *t* tests for continuous variables and chi-square test for categorical variables. Listwise deletion method will be adopted in cases of dropouts, if the lost values satisfy the criteria of the missing data completely at random; otherwise, multiple imputation data strategy will be used. The Kolmogorov-Smirnov and Levene tests will be used to analyze the normality and homogeneity of data, determining further statistical tests for detecting differences between groups (*P*<.05). A Student *t* test or Mann-Whitney *U* test will be employed for parametric or nonparametric analyses, respectively.

## Results

At this moment, 104 dyads have already been recruited. The parents or caregivers have responded the questionnaires, while children are being examined to determine their baseline oral health status. From August, 2019, dyads will be allocated to control or test groups. The parents or caregivers will receive the first electronic message after 15 days of allocation. The first results related to oral health status of children are expected to be obtained in November, 2019. The data collection will be performed during 12 months, between August 2019 and 2020.

## Discussion

Dental caries in deciduous teeth continue to affect the quality of life of children and their caregivers, regardless of their socioeconomic status, threatening the public health systems globally [28,29]. The prevalence of ECC ranges from 12% to 40%, with some developing regions reaching up to 98% [30,31]. Its incidence is approximately of 15,205 cases per 100,000 people, with millions of children diagnosed with the disease worldwide [11]. In 2016, the burden of untreated dental caries in deciduous teeth reached 6.59 disability-adjusted life-years for each 100,000 Brazilian preschoolers [32].

ECC is characterized by a multifactorial etiology, with a complex rampant progression that depends on the risk factors related to socioeconomic conditions such as low education levels, cariogenic diet, inadequate feeding practices, poor oral hygiene, and harmful daily habits under the responsibility of parents or caregivers [33-36]. The probability of the progression of this disease is high when there is no preventive or treatment interventions available [37]; however, the approach of treating ECC exclusively with dental restorative procedures is no longer considered appropriate. In this sense, the American Academy of Pediatric Dentistry recommends the following medical principles for controlling chronic noncommunicable diseases to reach better oral health outcomes [38,39] such as behavioral management focused on supplying specific needs of children and their caregivers [40]. Evidence supports a correlation of caregivers’ low health literacy skills with inadequate child health status [41,42], weak disease resolution, lack of medication adherence [43,44], difficult-to-follow professional instructions [45], and lower medical visit rates [46]. Then, the limited capacity of parents or caregivers in comprehending materials containing essential knowledge for the prevention of ECC may impact directly on the outcomes of health education [41].

Parents and caregivers seem to play an important role in the management of chronic diseases of their children [47,48], especially when these diseases affect children under 3 years, which is observed with ECC. Mobile technologies have become important resources to empower patients to be active on their own conditions through shared decision-making process [49-52]. Nevertheless, parents and caregivers could be less used to electronic devices and even less to download apps for their mobiles or tablets [47,53]. Hence, ECC-related information delivered via WhatsApp Messenger could be an alternative to engage parents or caregivers in a healthy lifestyle, contributing to a better patient-professional relationship. This app is a low-cost instant-messaging platform very popular in Latin America, and more specifically in Brazil, where 56% of the population uses it frequently [54]. According to Ojeda et al [55], WhatsApp Messenger demonstrated its effectiveness for contacting patients, independently of their education level, making it a reliable source of information and an easy way for patient-professional interaction. A previous study reported a significant reduction of dental plaque in adolescents after using WhatsApp Messenger as an adjuvant for the prevention of oral diseases [56]. However, to our knowledge, there is no evidence showing the effect of educational electronic messages sent to parents or caregivers on the prevention of ECC.

These outcomes will demonstrate the influence of educational mobile messages sent via WhatsApp Messenger on the different parameters of oral health status of children, such as biofilm accumulation, dental demineralization, gingival health, and sugar consumption. In addition, the effect of this study protocol on the levels of eHealth literacy and perceived self-efficacy of parents or caregivers will be elucidated. All aforementioned analyses will consider the effect of possible sociodemographic confounding factors, after stratifying and controlling participants according to eHealth literacy levels of parents or caregivers and the ICDAS index, age, and gender of children.

The evidence produced in this study can support the development of digital strategies to be applied in ECC preventive programs, considering their 3 main advantages: scalability, cost-effectiveness, and empowerment of laypersons for self-dental care.

## Appendix

Multimedia Appendix 1 Peer-review report from São Paulo Research Foundation.

## Abbreviations

CPITN: community periodontal index of treatment needs
ECC: early childhood caries
eHealth: electronic health
GSES: General Perceived Self-Efficacy Scale
ICDAS: International Caries Detection and Assessment System
RCT: randomized controlled trial
VPI: visible plaque index
