# Supplementary material for: The Effectiveness of Educational Mobile Messages for Assisting in the Prevention of Early Childhood Caries: Protocol for a Randomized Controlled Trial
Source: JMIR Res Protoc. 2019 Sep 3;8(9):e13656. doi: 10.2196/13656 (PMC6751091; doi:10.2196/13656)
Supplement: Multimedia Appendix 1 [file resprot_v8i9e13656_app1.pdf]

VISUALIZAÇÃO DE DESPACHO

**Processo**	2017/25899-7
**Linha de Fomento**	Programas Regulares / Bolsas / No País / Mestrado - Fluxo Contínuo
**Situação**	Em Execução
**Vigência**	01/05/2018 a 30/04/2020
**Beneficiário**	Matheus Lotto de Almeida Souza
**Responsável**	Thiago Cruvinel da Silva
**Vínculo Institucional do Processo**	Faculdade de Odontologia de Bauru/FOB/USP

Folha de Despacho para Reconsideração 001 - Mestrado

Resultado

Concedido

Datas do Despacho

**Emitido em :**	12/04/2018

Orçamento Consolidado

**Benefícios**	**Solicitado**		**Despacho**	
	**Valor (R$)**	**Valor (US$)**	**Valor (R$)**	**Valor (US$)**
Custeio				
Despesas de Transporte	0,00	0,00	0,00	0,00
Instalação	0,00	0,00	0,00	0,00
Reserva Técnica de Bolsa	4.917,96	0,00	4.917,96	0,00
Total	4.917,96	0,00	4.917,96	0,00
Bolsas				
Mestrado	49.179,60	0,00	49.179,60	0,00
Total	49.179,60	0,00	49.179,60	0,00
Total Geral	54.097,56	0,00	54.097,56	0,00

Dados de Execução

**Data Início**	01/05/2018
**Duração**	24 mês(es)
**Data Término**	30/04/2020
**Área de alocação de recursos**	Saúde
**Relatório Científico (Quantidade)**	2
**Relatório Científico (Datas)**	10/04/2019

	10/05/2020
**Ata de Defesa (Quantidade)**	1
**Ata de Defesa (Datas)**	15/11/2020
**Prestação de Contas (Quantidade)**	2
**Prestação de Contas (Datas)**	10/04/2019<br>10/05/2020
**Permite prorrogação da Bolsa?**	Não

Observações / Transcrições / Frases

Observações ao Responsável

Comunicamos que sua solicitação de bolsa, constante do processo acima referido, foi analisada e aprovada pela FAPESP.

Por favor, aguarde o email com as instruções para confirmação de interesse pela concessão.

Para conhecimento do conteúdo do despacho, por favor, acesse o Sistema SAGe (www.fapesp.br/sage), selecionando o item do menu Meus Processos>>Número do Processo e, em Mais Informações, a opção Despacho.

Com exceção de bolsa de Pós Doutorado, a transcrição do parecer está disponível exclusivamente para o orientador, sendo de sua responsabilidade escolher os trechos a serem compartilhados com o candidato.

Caso se aplique, o benefício de auxílio instalação deve ser objeto de Solicitação de Mudança a ser apresentada no momento do Aceite da Concessão. Por favor, verifique os critérios de elegibilidade e as instruções para apresentação no site da FAPESP, em http://www.fapesp.br/7771.

Para qualquer consulta ou comunicação sobre esta correspondência, por favor, use exclusivamente os serviços do "Converse com a FAPESP" em www.fapesp.br/converse.

Atenciosamente,

Carlos Henrique de Brito Cruz
Diretor Científico

PENDÊNCIAS:

1. Comprovante de conclusão da graduação.
2. Comprovante de matrícula no mestrado.

Frases para o Responsável

Não há frases associadas.

Transcrição de Parecer para o Responsável

ANÁLISE GERAL DA PROPOSTA - A FAPESP denomina "Proposta" o conjunto de três partes a serem avaliadas, composto por: 1. Projeto de Pesquisa; 2. Histórico Escolar e Acadêmico do Candidato; e, 3. Histórico de Pesquisa do Orientador. Por favor preencha este item depois de preencher o restante do formulário.

Projeto apresentado agora contém metodologia mais detalhada e como avaliado anteriormente apresenta um tema relevante. O histórico escolar do aluno é bom e o acadêmico é muito bom, demonstrando interesse no campo da pesquisa. O histórico de Pesquisa do Orientador é excelente, pois tem publicado com regularidade em Revistas de impacto e tem concretizado orientações.

Por favor, analise o PROJETO DE PESQUISA APRESENTADO seguindo o roteiro abaixo

----

Definição e pertinência dos objetivos.

Os 2 objetivos são pertinentes segundo argumentos apresentados na introdução e metodologia: (1) a investigação de temas e conteúdos relacionados às dúvidas mais frequentes sobre a doença e (2) a avaliação da eficácia do envio de mensagens de texto via celular aos pais ou responsáveis como método auxiliar para a prevenção da CPI em crianças.

Importância da contribuição pretendida para a área do conhecimento em que o projeto proposto se insere.

Como a prevalencia e gravidade da CPI são de grande impacto, inclusive para a dentição permanente, o uso de metodologias preventivas simples e acessíveis são relevantes.

Fundamentação científica e os métodos empregados.

Foram adicionados/esclarecidos alguns itens que não haviam sido abordados na 1a versão, adotando como referência Bardin,2011, com avaliação do material por 2 pesquisadores.Foram detalhados também os passos para o treinamento e calibração dos examinadores.Foram detalhados os índices a serem utilizados.

Adequação do projeto a um programa de mestrado e viabilidade de sua execução no prazo previsto.

O tema e cronograma apresentados são compatíveis a um programa de Mestrado e viáveis no tempo existente.

Caso se trate de um candidato que já tenha iniciado o Mestrado, avalie o andamento do projeto de pesquisa e a viabilidade de sua execução no prazo previsto.

O candidato finalizou seu curso de Graduação em 7/12/2017, sendo assim ainda não possui atividades na Pós Graduação.

Conclusão sobre a análise do Projeto de Pesquisa apresentado. (Preenchimento obrigatório)

[ ] Excelente
[X] Muito boa
[ ] Muito boa, com algumas deficiências facilmente sanáveis
[ ] Boa
[ ] Boa com deficiências
[ ] Regular
[ ] Com sérias deficiências

Por favor, avalie o HISTÓRICO ESCOLAR E ACADÊMICO DO CANDIDATO - As bolsas de Mestrado se destinam a estudantes com bom desempenho acadêmico, avaliado principalmente pelo histórico escolar de graduação e pós-graduação. A concessão a estudantes com histórico escolar irregular, exibindo um padrão de reprovações ou aprovações com nota mínima, é possível apenas em circunstâncias excepcionais à vista de outras evidências sobre o potencial acadêmico do candidato, como por exemplo, projeto bem sucedido de Iniciação Científica.

Histórico Escolar do candidato.

O candidato apresentou média de 7,4, sendo classificado como 28o. na turma de 50 alunos.
Considero como bom o histórico escolar da graduação.

Histórico Acadêmico do candidato (participação em projetos de pesquisa, bolsas anteriores, publicações científicas, premiações, etc.).

O candidato apresenta um histórico muito bom de atividades académicas, tendo 2 artigos publicados e 9 trabalhos apresentados em Congressos com 5 resumos publicados. Sendo assim, participou de Congressos e obteve 3 menções honrosas.

Conclusão sobre a análise do Histórico Escolar e Acadêmico do Candidato. (Preenchimento obrigatório)

[ ] Excelente
[X] Muito bom
[ ] Bom
[ ] Regular
[ ] Com sérias deficiências

Por favor, avalie o HISTÓRICO DE PESQUISA DO ORIENTADOR

Qualidade, regularidade e importância da produção científica e/ou tecnológica, com destaque para a produção recente. Elementos importantes para essa análise são: lista de publicações em periódicos com seletiva política editorial; livros ou capítulos de livros; patentes em que figure como inventor; outros instrumentos de propriedade intelectual; resultados de pesquisa efetivamente transferidos e adotados por empresas ou pelo governo; e outras informações que possam ser relevantes.

Da lista dos 10 artigos publicados de maior relevância, há 3 com tema relacionado a presente proposta. O pesquisador apresenta 44 publicações, participação em projetos e obtenção de financiamentos.

Experiência e competência demonstradas na liderança de projetos de pesquisas relacionados ao tema da proposta em análise

Obteve 2 prêmios relacionados a trabalhos apresentados com o tema desta proposta.

Analise a capacidade demonstrada para formar pesquisadores, com destaque para a atividade recente de orientação.

Orientou 2 Mestrados e 7 iniciações cientificas com bolsa.

Analise os resultados obtidos pelo orientador com financiamentos anteriores da FAPESP.

Tem um auxilio vigente com data de concessão de 2015.

Analise a disponibilidade para orientação considerando o regime de trabalho e o número total de estudantes sob a sua orientação.

Ha disponibilidade para execução deste trabalho, considerando experiencia previa e dedicação exclusiva.

Número atual de orientandos.

6 IC
1 MS
2 DR

Regime de trabalho.

Dedicação Exclusiva.

Outras considerações sobre a produção científica, tecnológica e acadêmica do orientador, relevantes para a avaliação da viabilidade da proposta.

Como descrito agora na reconsideração foi autor correspondente publicando 06 artigos de pesquisa nos últimos 12 meses,, sendo três em revistas Qualis A1 (Caries Research, PloS One e Journal of Medical Internet Research), um em revista Qualis A2 (Lasers in Medical Science) e dois em revistas Qualis B1 (PeerJ e Photodiagnosis and Photodynamic Therapy), dos quais quatro pertencentes à presente linha de pesquisa. A produção foi advinda da orientação de seis alunos de iniciação científica, dois mestrandos e dois doutorandos recém-ingressados no PG. Ou seja, demonstra um papel dinâmico no campo da pesquisa e orientação.

Conclusão sobre a Análise do Histórico de Pesquisa do Orientador. (Preenchimento obrigatório)

[X] Excelente
[ ] Muito Bom
[ ] Bom
[ ] Regular
[ ] Com sérias deficiências

DEFICIÊNCIAS NOTADAS NA PROPOSTA (Se algum dos itens abaixo for assinalado, as razões devem ser explicitadas no quadro correspondente do formulário).

Sobre o Projeto

[ ] Projeto com objetivos mal definidos, excessivos ou incongruentes
[ ] Projeto com objetivos excessivamente limitados
[ ] Projeto pouco original
[ ] Contribuição pouco significativa para a área de conhecimento
[ ] Fundamentação científica insuficiente/metodologia inadequada
[ ] Inadequado para um programa de Mestrado
[ ] Viabilidade de execução questionável
[ ] Andamento do projeto é inadequado, comprometendo a viabilidade de execução do mesmo

----

Sobre o Histórico Escolar e Acadêmico do Candidato

[ ] Histórico escolar deficiente
[ ] Candidato com potencial não evidenciado

Sobre o histórico de Pesquisa do Orientador

[ ] Produção científica ou tecnológica que não atesta significativo rendimento da atividade de pesquisa
[ ] Experiência insuficiente na área de pesquisa em que se insere o projeto, podendo comprometer a sua viabilidade
[ ] Capacidade de orientação não evidenciada
[ ] A produção científica ou tecnológica resultante de auxílios anteriores é insatisfatória

----

Outras deficiências

[ ] Mestrado iniciado há mais de ano
[ ] Outras

Justificar "Outras Deficiências"

**Frases para Termo de Outorga**
Não há frases associadas.

Orçamento Detalhado - Quadros Resumos

**Reserva Técnica de Bolsa - Solicitado**	
**Percentual para Reserva Técnica (País)**	10,00 %
**Percentual para Reserva Técnica (Exterior)**	0,00 %
**Dólar FAPESP**	3,50
**Valor da Reserva Técnica (R$)**	4.917,96
**Valor da Reserva Técnica (US$)**	0,00

**Reserva Técnica de Bolsa - Despacho**	
**Percentual para Reserva Técnica (País)**	10,00 %
**Percentual para Reserva Técnica (Exterior)**	0,00 %
**Dólar FAPESP**	3,50
**Valor da Reserva Técnica (R$)**	4.917,96
**Valor da Reserva Técnica (US$)**	0,00

**Bolsa**		
**Beneficiário**	Matheus Lotto de Almeida Souza	
**Modalidade**	Solicitado<br>Mestrado - MS-1	Despacho<br>Mestrado - MS-1
**Duração**	24 mês(es)	24 mês(es)
**Data Início**	10/03/2018	01/05/2018
**Moeda**	R$	R$
**Valor Unitário**	1.988,10	1.988,10
**Valor Total das Mensalidades**	49.179,60 reais	49.179,60 reais
**Justificativa**		

**Bolsas do Beneficiário**
Nenhuma bolsa encontrada.

Orçamento Detalhado - Itens de despesa

**Reserva Técnica de Bolsa**	
**Percentual para Reserva Técnica (País)**	10,00 %
**Percentual para Reserva Técnica (Exterior)**	0,00 %

**Dólar FAPESP**	3,50
**Valor da Reserva Técnica (R$)**	4.917,96
**Valor da Reserva Técnica (US$)**	0,00

**Bolsa**	
**Modalidade**	Mestrado - MS-1
**Quantidade mensalidades 1° nível**	12 mês(es)
**Beneficiário**	Matheus Lotto de Almeida Souza
**Data Início**	01/05/2018
**Duração**	24 mês(es)
**Moeda**	R$
**Valor Total das Mensalidades**	49.179,60 reais
**Justificativa**	
